# Whale phylogeny and rapid radiation events revealed using novel retroposed elements and their flanking sequences

**DOI:** 10.1186/1471-2148-11-314

**Published:** 2011-10-27

**Authors:** Zhuo Chen, Shixia Xu, Kaiya Zhou, Guang Yang

**Affiliations:** 1 Jiangsu Key Laboratory for Biodiversity and Biotechnology, College of Life Sciences, Nanjing Normal University, Nanjing 210046, China

## Abstract

**Background:**

A diversity of hypotheses have been proposed based on both morphological and molecular data to reveal phylogenetic relationships within the order Cetacea (dolphins, porpoises, and whales), and great progress has been made in the past two decades. However, there is still some controversy concerning relationships among certain cetacean taxa such as river dolphins and delphinoid species, which needs to be further addressed with more markers in an effort to address unresolved portions of the phylogeny.

**Results:**

An analysis of additional SINE insertions and SINE-flanking sequences supported the monophyly of the order Cetacea as well as Odontocete, Delphinoidea (Delphinidae + Phocoenidae + Mondontidae), and Delphinidae. A sister relationship between Delphinidae and Phocoenidae + Mondontidae was supported, and members of classical river dolphins and the genera *Tursiops *and *Stenella *were found to be paraphyletic. Estimates of divergence times revealed rapid divergences of basal Odontocete lineages in the Oligocene and Early Miocene, and a recent rapid diversification of Delphinidae in the Middle-Late Miocene and Pliocene within a narrow time frame.

**Conclusions:**

Several novel SINEs were found to differentiate Delphinidae from the other two families (Monodontidae and Phocoenidae), whereas the sister grouping of the latter two families with exclusion of Delphinidae was further revealed using the SINE-flanking sequences. Interestingly, some anomalous PCR amplification patterns of SINE insertions were detected, which can be explained as the result of potential ancestral SINE polymorphisms and incomplete lineage sorting. Although a few loci were potentially anomalous, this study demonstrated that the SINE-based approach is a powerful tool in phylogenetic studies. Identifying additional SINE elements that resolve the relationships in the superfamily Delphinoidea and family Delphinidae will be important steps forward in completely resolving cetacean phylogenetic relationships in the future.

## Background

**Figure 1:**
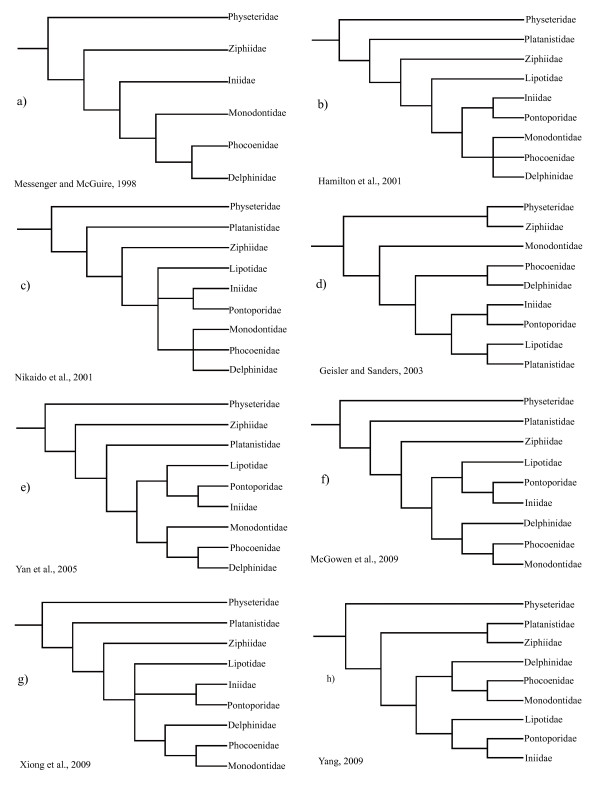
**Alternative hypotheses of phylogenetic relationships among the major odontocete lineages as obtained from morphological and molecular sequence data**.

**Figure 2:**
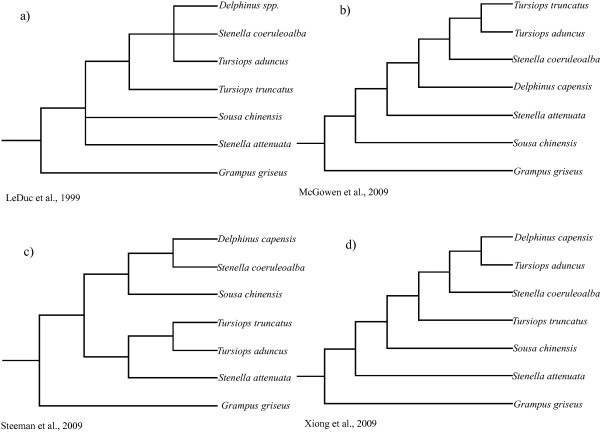
**Recent hypotheses of the interrelationships of *Grampus-Sousa-Delphinus--Tursiops-Stenella *complex**. The original phylogenies were pruned to include only species used in the current study.

Extant cetaceans (whales, dolphins and porpoises), which consist of approximately 89 species in 14 families, are ecologically diverse, ranging from coastal to oceanic and from tropical to polar waters [1]. The order Cetacea has traditionally been divided into two highly distinct suborders: Mysticeti (the filter-feeding baleen whales) and Odontoceti (the echolocating toothed whales). Cetaceans differ dramatically from other mammals in terms of morphology, behavior and ecology, representing one of the most fascinating evolutionary transitions within vertebrates. The phylogeny of Cetacea has long attracted interest of evolutionary biologists and has been investigated using both morphological (including fossil) and molecular data [2-33]. Some of the issues have been well resolved including the monophyly of Cetacea [5,12-17,19-22] and its sister relationship with Hippoptamidae [10,12,13,22-24]. However, these studies left unresolved issues: 1) the phylogenetic relationships of some major cetacean lineages; 2) the systematic status and phylogenetic position of some taxa such as the Ganges River dolphin or susu (*Platanista gangetica*) and the now nearly extinct Yangtze river dolphin or Baiji (*Lipotes vexillifer*), as well as those between the three delphinoid families: Monodontidae (narwhals and belugas), Phocoenidae (porpoises) and Delphinidae (dolphins) (Figure 1). The phylogenetic relationships among the various river dolphin genera (*Inia*, *Pontoporia*, *Platanista*, *Lipotes*) remain controversial, despite that a variety of studies have been conducted using a diverse array of systematic markers [12,17,31,33], even in large concatenations of data [10]. The now nearly extinct *Lipotes *has been difficult to classify especially with respect to *Inia *and *Pontoporia *[12,31]. Additionally, the position of *Platanista *at the base of Odontoceti was unstable, with conflicting evidence coming from morphology, mtDNA, and nuclear DNA (reviewed in [10]). In addition to these conflicts, previous phylogenetic hypotheses disagreed with one another in revealing relationships and diversity of the species within Delphinidae, especially within the *Sousa-Delphinus-Tursiops-Stenella *complex (Figure 2). In this complex, *Tursiops truncatus *(bottlenose dolphin) was long considered as the single species in the genus *Tursiops*, but recently two species, *T. truncatus *and *T. aduncus*, have been recognized as valid for this genus [34-36]. LeDuc et al. [34] suggested that *T. aduncus *was more closely related to the striped dolphin (*Stenella coeruleoalba*) than to the congener *T. truncatus *based on cytochrome *b *analysis. This is contrasted with morphological and other molecular evidence supporting *Tursiops *and *Stenella *as monophyletic genera [10,11,35].

SINEs (short interspersed elements) have been proposed as perfect molecular markers for studies of systematics, phylogenetics, evolution, and population biology, etc. [16,22,23,31,32,37-47]. They have been successfully applied to resolve phylogenetic relationships among various groups at different taxonomic ranks [31,32,37,39-42,44]. SINEs are one of the major classes of retroposons that are dispersed throughout eukaryotic genomes. They are nonautonomous retroposons lacking the machinery to replicate themselves and they propagate in the genome via cDNA intermediating and are reintegrated into the host genome by retroposition [48-51]. Integration of a SINE sequence at a specific site in the genome is irreversible, and its target site is chosen almost at random [52]. To date, no mechanism has been described for the reversal of retroposon integration, and it is highly unlikely that the same type of retroposon would be integrated into the same genomic locus independently in different lineages [53]. SINEs, which are shared by some taxa but missing from the genomes of others, are ideal shared, derived phylogenetic characters at the molecular level [22,31,32,37-47,51-56]. Thus, a SINE sequence found at an orthologous locus in two or more lineages can be regarded as evidence for synapomorphy.

Nikaido and his colleagues pioneered the use of SINE insertions to address the relationships among cetaceans and other orders of mammals as well as to address relationships among both mysticetes and odontocetes [16,22,31,32,44]. For example, they examined 25 informative SINE insertions to support the monophyly of toothed whales and the paraphyly of river dolphins [31]. However, the interrelationships among some cetacean lineages, especially three families within Delphinoidea (i.e. Delphinidae, Phocoenidae and Mondontidae), were not well resolved with SINE markers, although their analysis of the SINE-flanking sequences supported the sister group relationship of Monodontidae and Phocoenidae with the exclusion of Delphinidae.

Thus, the main objectives of the present study are to: 1) address some of the remaining problematic areas of the cetacean phylogenetic tree through the analysis of additional SINE insertions and flanking sequences, and 2) utilize flanking sequences of 12 retroposed elements to estimate divergence times associated with the cetacean radiation. Identifying additional SINE elements that resolve the relationships within superfamily Delphinoidea and family Delphinidae will be important steps forward in completely resolving cetacean phylogenetic relationships in the future.

## Results

### Phylogenetic relationships

A total of 219 insertion loci were identified from random sequencing of genomic DNA from the Indo-Pacific bottlenose dolphin, screening genomic libraries from five species (i.e. long-beaked common dolphin, striped dolphin, Indo-Pacific humpbacked dolphin, Risso's dolphin, and finless porpoise), and screening the genome sequence of the common bottlenose dolphin. After eliminating loci that failed to amplify in all taxa (118 loci), were difficult to decipher (1 locus), and were present in all taxa (36 loci), 64 loci proved phylogenetically informative (Additional file 1 and 2).

**Figure 3:**
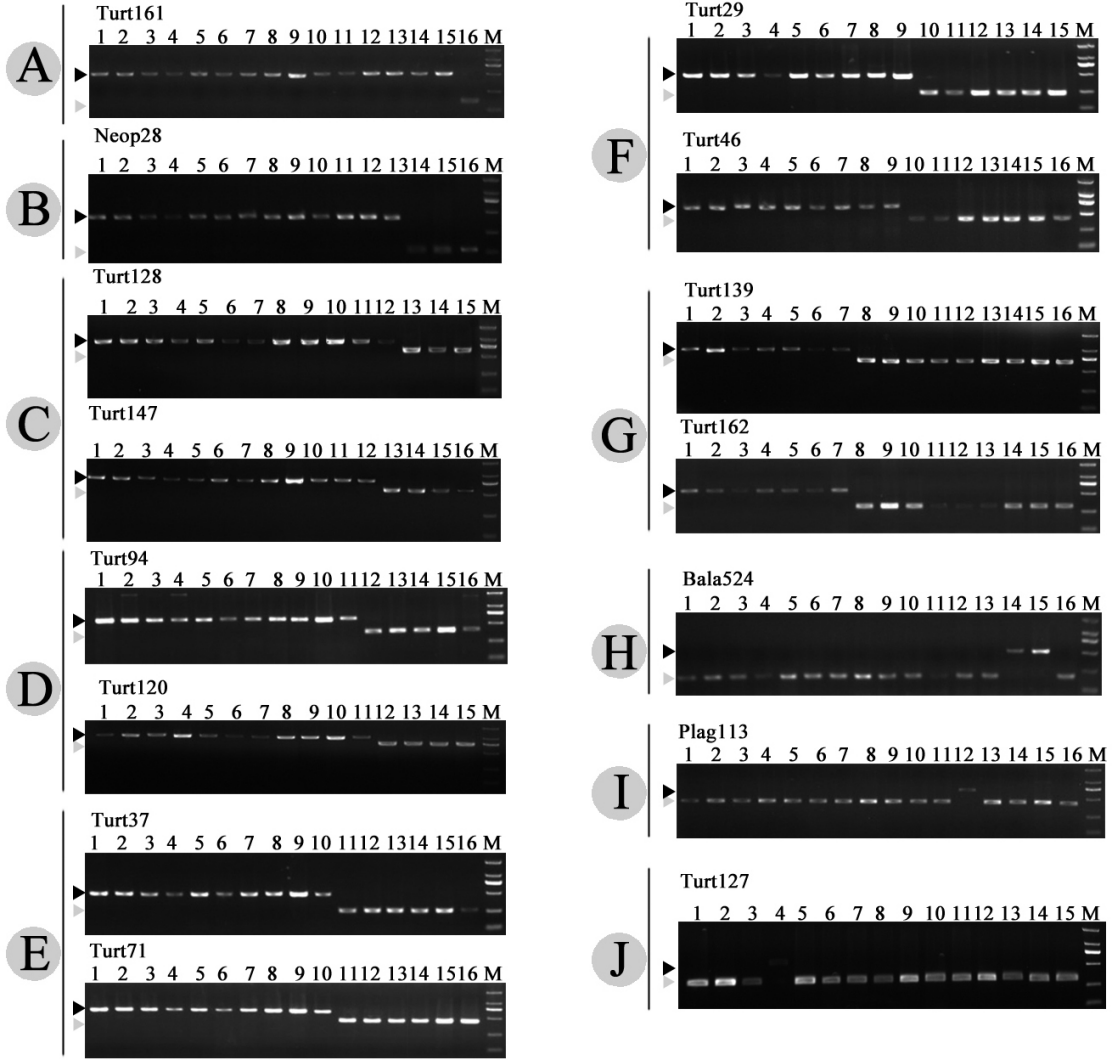
**Electrophoretic gel patterns of PCR products for 15 representative SINE loci**. All loci analyzed in this study are shown in Additional file 1. Bands indicating the presence of the SINE are shown by black arrowheads, whereas gray arrowheads show those that indicate SINE absence. Loci are assigned alphabetically from A to J according to the clade on the phylogenetic tree shown in Figure 4. The species are numbered as follows: 1, Striped dolphin; 2, Risso's dolphin; 3, Indo-Pacific bottlenose dolphin; 4, Common bottlenose dolphin; 5, Long-beaked common dolphin; 6, Chinese white dolphin; 7, Pantropical spotted dolphin; 8, Beluga; 9, Finless porpoise; 10, Yangtze River dolphin; 11, Ginkgo-toothed beaked whale; 12, Ganges River dolphin; 13, Pygmy sperm whale; 14, Omura's whale; 15, Common minke whale; 16, hippopotamus.

**Figure 4:**
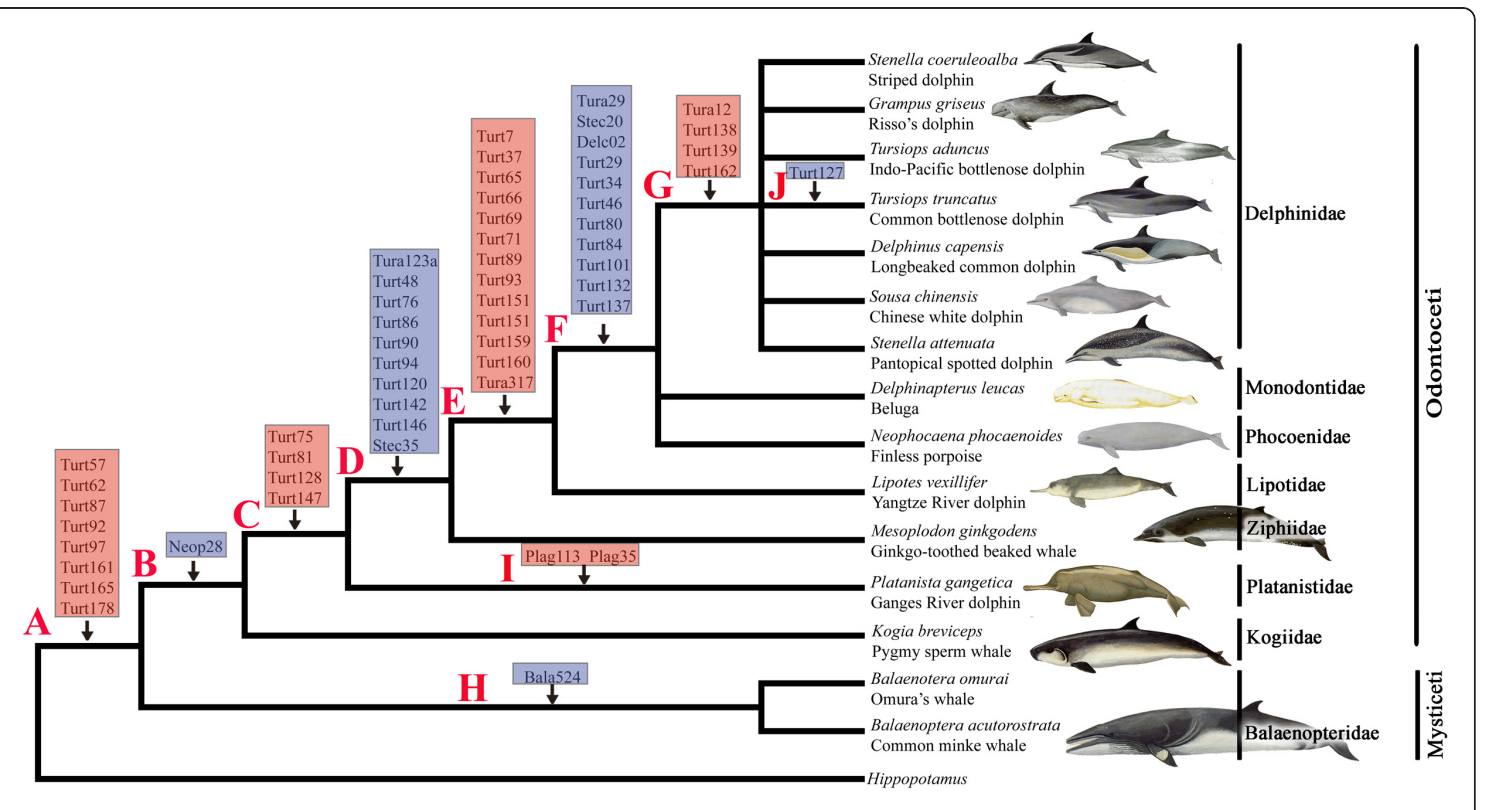
**Phylogenetic relationships of the major lineages of Cetaceans reconstructed using retroposon insertion data shown in Additional file **2. Closed vertical arrowheads denote insertions of retroposons into each lineage. All loci mapped onto the tree were newly isolated and characterized in the present study. Each clade is named alphabetically from A to J. Cetacean families are delimited by vertical lines to the right of the tree along with representative members.

Figure 3 shows the PCR patterns of 15 representative SINE loci in cetacean clades of A-J. Eight newly isolated SINE loci are present in all cetaceans but not in the hippopotamus, supporting the monophyly of the order Cetacea (clade A in Figures 3 and 4 and Additional file 1). Clade B represented the monophyly of the suborder Odontocete (toothed whales), which was supported by one independent locus Neop28 (Figures 3 and 4 and Additional file 1). Furthermore, we also elucidated the order from which toothed whales diverged. The sister relationship between sperm whales and the other toothed whales was supported by one SINE insertion Neop28 (Figures 3 and 4 and Additional file 1). The Ganges River dolphins and the remaining toothed whales formed a monophyletic group supported by the presence of four SINE insertions (clade C in Figures 3 and 4 and Additional file 1). The sister relationship between beaked whales and Yangtze River dolphin + Delphinoidea (Delphinidae + Phocoenidae +Mondontidae), as well as a sister relationship of the latter two families were supported by ten and thirteen SINE loci respectively (clade D and E in Figures 3 and 4 and Additional file 1). Finally, the monophyly of the superfamily Delphinoidea was supported by eleven informative loci (clade F in Figures 3 and 4 and Additional file 1). Within the superfamily Delphinoidea, the differentiation between Delphinidae and other two families was clearly suggested with four SINE insertions (clade G in Figures 3 and 4 and Additional file 1). Four SINE insertions indicate clades from H to J (Figures 3 and 4, Additional file 1). For example, the locus Plag35 and Plag113 indicated two species-specific integrations for the Ganges River dolphins, whereas the locus Turt127 indicated a species-specific insertion for the Common bottlenose dolphin.

**Figure 5:**
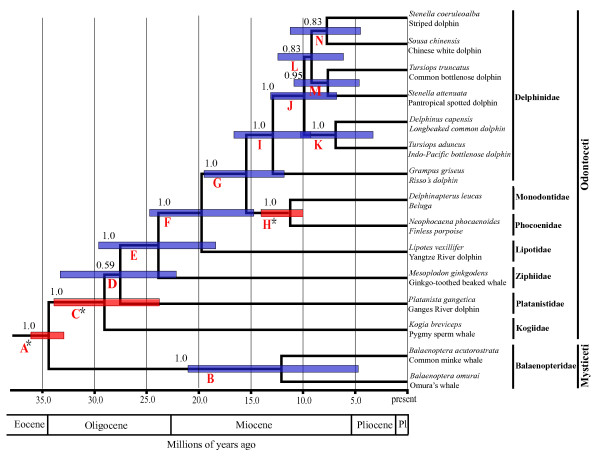
**Time-calibrated cetacean phylogeny derived from BEAST using the flanking regions of 12 retroposed elements**. Numbers above the clades represent Bayesian posterior probabilities. Clade letters are identical to those in Table 1. Red boxes indicate nodes for which a prior calibration constraint distribution was used and blue boxes indicate divergence dates estimated without prior calibration constraints for that node. The bounds of the boxes correspond to the 95% highest posterior density (HPD) of each node.

Figure 5 shows the cetacean relationships inferred from Bayesian analysis of the 3, 974 sites of SINE-flanking sequences. The topology supported the monophyly of Odontoceti (toothed whales), with a posterior probability of 1.00. The basal divergence within odontocetes is between the physeteroids (with the pygmy sperm whale as the representative) and a clade (PP = 1.0) of remaining odontocete species. The sister relationship between Platanistidae (Indian River dolphins) and other dolphins and porpoises was weakly supported (PP = 0.59), whereas the relationship between Ziphiidae (beaked whales) and Lipotidae (Yangtze River dolphin) + Delphinoidea (Delphinidae + Phocoenidae +Mondontidae) was well supported with PP = 1.0, and the support for the sister relationship of the latter two families was significant (PP = 1.0). The oceanic dolphins and porpoises formed a clade (PP = 1.0), with a basal divergence between monophyletic Delphinidae (PP = 1.0) and a sister relationship of Phocoenidae (porpoises) and Monodontidae (narwhals and belugas) (PP = 1.0). Within the Delphinidae, the Risso's dolphin (*G. griseus*) was the sister group to the remaining delphinids, whereas the remaining delphinids were subdivided into two clades: one well supported clade *T. aduncus *+ *D. capensis *(Figure 5, clade K; PP = 1.0), and the other weakly supported clade ((*Sousa chinensis + St. coeruleoalba*) + (*T. truncatus *+ *St. attenuata*)) (Figure 5, clade L; PP = 0.83). As revealed in previous studies, two species of *Tursiops *(*T. truncates *and *T. aduncus*) and two species of *Stenella *(*St. coeruleoalba *and *St. attenuata*) did not form respective monophyletic clades, which suggested that both genera are not monophyletic.

### Divergence time estimation

All estimated divergence dates for nodes with labels from A to N in Figure 5 were presented in Table 1. The split between Mysticeti and Odontoceti was estimated to have occurred in the Late Eocene, shortly before the appearance of the first documented fossil mysticete *Llanocetus denticrenatus *(~34.2 MYA) (Figure 5). Radiation of the major clades of Odontocetes (Physeteroidea, Platanistidae, Ziphiidea, Lipotidae, Delphinoidea) dated from 15.55 to 29.05 MYA (Figure 5 and Table 1), suggesting a rapid early radiation within the major Odontocete lineages. These estimates are close to and at some degree later than previous estimates which were primarily based on mitochondrial DNA sequences and other markers [10,12,19,31]. The divergence of the three extant delphinoid families took place in the Middle Miocene, whereas the radiation of the crown Delphinid lineages appeared to occur in the Middle Miocene, while the *Sousa-Delphinus-Tursiops-Stenella *complex may have a recent divergence in the Middle-Late Miocene and Pliocene.

**Table 1:** Divergence times of lineages analyzed in this study, estimated from Bayesian phylogenetic analyses of the flanking regions of 12 retroposed elements using a lognormal relaxed molecular clock.

Clade	Age	Lower 95% HPD	Upper 95% HPD
A*	34.40	33.52	36.09
B	12.09	4.75	21.03
C*	29.05	23.79	33.90
D	27.53	22.18	33.30
E	23.88	18.40	29.62
F	19.75	14.73	24.73
G	15.55	11.81	19.45
H*	11.39	10.02	14.02
I	12.90	9.25	16.63
J	9.89	6.78	13.11
K	6.88	3.29	10.25
L	9.18	6.14	12.42
M	7.63	4.62	10.88
N	7.81	4.49	11.24

Clade letters refer to those shown in Figure 5. The asterisk indicates that this clade was constrained in the phylogenetic analysis. HPD = highest posterior density. Units are in million years, MY.

### Anomalous PCR amplification patterns of retroposon insertions in cetaceans

**Figure 6:**
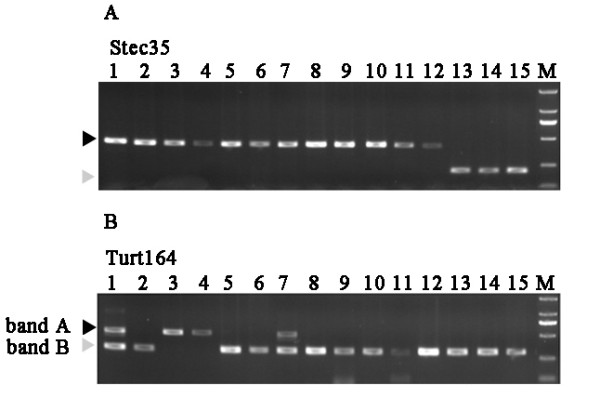
**Potential confounding SINE insertions**. Two samples of incongruent loci are shown. Picture 6A shows a near-parallel insertion event occurring at locus Stec35. Picture 6B is the agarose gel electrophoresis result of Turt164 from 15 cetacean samples. It is polymorphic in the two species of the genus *Stenella*. The species are numbered as in Figure 3.

Although the vast majority of SINE insertions in our study supported a single most parsimonious tree, two anomalies in the present SINE analysis of phylogenetics remain noteworthy. At the locus Stec35, it was present in the Ganges River dolphins, based on preliminary analysis of the agarose gel electrophoresis. However, further analysis of the DNA sequences indicated that a different SINE insertion has occurred near the insertion Stec35 locus (68-bp distance between the two loci) (Figure 6A and Additional file 3). This indicated that the locus Stec35 was absent in the genome of the Ganges River dolphins, instead, there was a novel species-specific insertion and we tentatively named it Plag35, owing to its discovery only in *Platanista gangetica*.

The second anomaly came from the locus Turt164. *St. attenuate *and *St. coeruleoalba *exhibited the typical heterozygous profile consisting of the insertion amplicon (band A) and the lack of insertion PCR product (band B) (Figure 6B) at this locus, while nearly all other species examined (exclusive of *T. truncatus *and *T. aduncus*) amplified a single amplicon of band B corresponding to the lack of insertion allele (Figure 6B). In contrast, *T. truncatus *and *T. aduncus *generated the usual single band A of homozygote for the insertion allele. To confirm this polymorphic amplification, four more *T. truncatus *and *T. aduncus *individuals were examined and they all generated the same single band A. In order to investigate this interesting scenario, different amplicon types (*i.e*., band A and band B) were isolated, cloned and sequenced (see Methods). As shown in Additional file 4, the only difference between the sequence of amplicon A and B in both *St. attenuate *and *St. coeruleoalba *is the lack of a SINE element in B.

## Discussion

### Phylogeny of Odontoceti and its Oligocene radiation

Relationships among odontocete families obtained in the present study were broadly congruent with most previous molecular and morphological hypotheses [5,7,8,12,13,19,20,29,31,33,57-65]. For example, the monophyly of Odontoceti and the sister relationship of Physeteroidea to all other extant odontocetes (Figures 4 and 5), supported the SINE analysis of Nikaido et al. [31] and was compatible with the morphological evidence [29]. The grouping of Ziphiidae (beaked whales) with Delphinida to the exclusion of Platanistidae and Physeteroidea (clade D in Figures 3 and 4 and Additional file 1), was concordant with previous SINE insertion analyses [31], as well as the SINE-flanking sequence analysis in the present study (Figure 5).

The grouping of the four genera of 'river dolphins' in family Platanistidae or superfamily Platanistoidea [66] has long been challenged by both morphologists and molecular systematists [5,7,19,31,33,60,61,67-69], and instead conflicting relationships of the four major river dolphin clades have been proposed (Figure 1). Although the lack of *Inia *and *Pontoporia *in the present study made it difficult to discern the phylogeny of river dolphins, the present finding that *Platanista *has no direct affinity with *Lipotes *clarifies that river dolphins are an artificial rather than a natural group, which is consistent with many previous molecular studies [7,31,33,63,69,70].

Our estimates of divergence times suggested that the common ancestors of extant cetaceans occurred in the Late Eocene Epoch, prior to approximately 34.40 (33.52-36.09; 95% highest posterior density) MYA (Figure 5 and Table 1), slightly younger than several previous estimates [10,11,19,31,71], but conflicted dramatically with the Early Eocene split around 50 MYA proposed by Cassens et al. [33] based on only one delphinid calibration. The present estimate accorded closely with the earliest known fossil crown cetacean, the archaic mysticete *Llanocetus denticrenatus *(~34.2) [72]. In addition, the present study estimates divergence of the major Odontocete lineages such as Physeteroidea, Platanistidae, Ziphiidea, Lipotidae and Delphinoidea occurred primarily in the Early Oligocene and Early Miocene (Figure 5 and Table 1). Climate change from greenhouse to icehouse which occurred in the Late Eocene to Early Oligocene [73,74] might have played an important role in the cetacean radiation. During that period, atmospheric CO_2 _level decreased, and the polar ice caps expanded rapidly, Southern Ocean upwelling and ocean productivity increased [75-78], which may explain the radiation of cetaceans [10-12,31]. Early representatives of cetacean fossils including Ferecetotherium, Waipatia and Kentriodontidae were present in the Late Oligocene, demonstrating that these lineages were diverged during this time period [79-82].

### Interrelationship within Delphinoidea and rapid divergence of Delphinidae

The interrelationships among the three families within Delphinoidea were disputed and several alternative branching patterns were proposed [2,5,7,17,19,20,29,31,33,64,65,70,83]. While several morphological and molecular studies agreed that a close relationship existed between Delphinidae and Phocoenidae [2,7,17,29], other molecular analyses supported the sister relationship of Monodontidae and Phocoenidae [12,13,19,20,33,65,70,83]. Besides these hypotheses, an unresolved relationship between the three families was mentioned in some studies [5,29,31,64]. In the present study, the differentiation between Delphinidae and other two families was suggested with four SINE insertions, while no SINE was found to suggest the divergence between Phocoenidae and Mondontidae. However, SINE flanking-sequences analysis here further resolved the relationship among three Delphinoidea families (Delphinidae + (Monodontidae + Phocoenidae)), which was the same as those revealed in Waddell et al. [83], Nishida et al. [8,65] and May-Collado and Agnarsson [70]. Within Delphinoidea, the divergence between Phocoenidae and Monodontidae was estimated at 11.39 (10.02-14.02; 95% highest posterior density) MYA (Figure 5 and Table 1), which are close to and at some degree later than previous analyses [10,12,19,84], but are much younger than Nikaido et al. [31], which predicted the divergence at 20 (17-23) MYA on the basis of SINE flanking sequences using the calibration date (55 Myr) for the separation of Cetacean from the hippopotamus based on the relaxed clock of cytochrome *b *data (lacking fossil calibration). Our result is consistent with the age of the oldest representative fossil, the late Miocene phocoenid *Salumiphocaena stocktoni *[80].

Of the Delphinidae species examined, the sister relationship of *Grampus griseus *and *Sousa-Delphinus-Tursiops-Stenella *complex [10-13,34,70] was confirmed by SINE-flanking sequences analysis with a posterior probability of 1.00 (Figure 5). Within *Sousa-Delphinus-Tursiops-Stenella *complex, it was supported the closest affinity between *Sousa *and *Stenella coeruleoalba*, with *T. truncatus *and *S. attenuata *as their sister clades, then they cluster with a clade of *D. capensis *and *T. aduncus*. This is in contrast with Caballero et al. 's [9] and McGowen et al. 's [10] suggestion of the basal position of *Sousa *among delphinine, the alliance of *Sousa *with *Stenella *and *Delphinus *[11], or the alliance of *Sousa *with *Steno *[85]. Further, the sister relationship of *T. aduncus *and *D. capensis *obtained in the present study with a posterior probability of 1.00 was well congruent with the studies based on Mt genomes [12]. Obviously, the present SINE flanking sequence data rejected the monophyly of genera *Tursiops *and *Stenella *[79,84,86-88], although some branches were not supported by high posterior probability (Figure 5). The delphinids was estimated to radiate in the Middle-Late Miocene and Pliocene, with branch events taking place within a narrow time frame (3-6 MYA) (Figure 5 and Table 1). Unfortunately, no SINE insertion was identified to solve the relationship within Delphinidae and especially within *Sousa-Delphinus-Tursiops-Stenella *complex, and more SINEs are necessary to solve this problem.

### Anomalous events in SINE-based phylogenetic analysis

Several potential anomalous SINE intertion events were revealed in the present study (Figure 6). These anomalies may have been brought about through near-parallel insertions, lineage sorting, and paralogous insertions, as discussed in previous studies [47].

### A. Parallel insertion

According to Ray et al. [47], near-parallel insertion meant that a secondary independent SINE was inserted into a site near the insertion originally being studied. To detect whether this is the case in cetaceans, we sequenced and analyzed the insertions. At locus Stec35, the original insertion was not found in the Ganges River dolphins, while an additional independent insertion was found to occur near the first insertion (68-bp interval between them) (as shown in Additional file 3).

### B. Anomalous PCR amplification patterns of Turt164: Paralogous insertion, incomplete lineage sorting, or introgressive hybridization?

Turt164 is another interesting SINE that appeared to be polymorphic (Figure 6B). For example, *St. attenuata *and *St. coeruleoalba *exhibited the typical heterozygous profile consisting of the insertion amplicon (band A) and the lack of insertion PCR product (band B), whereas a single PCR amplicon (band A or band B) was found in other representative species examined in the present study (Figure 6B).

Paralogous insertion [47,89] might be a potential interpretation of this anomalous phenomenon. Only one band (band A) was amplified from the genus *Tursiops*, a scenario that can be interpreted as segmental duplications occurred around the locus Turt164 of genus *Stenella*. Further studies including more samples of *Stenella *species should be performed to confirm this interpretation.

Incomplete lineage sorting [44,90] may also be an alternative cause. Rapid speciation might occur in the common ancestor of genera *Tursiops *and *Stenella *[10-13,79] and Turt164 inserted into their genome during a short period. This insertion might have been fixed in genus *Tursiops*, but not in genus *Stenella *because of incomplete lineage sorting. However, because only a small number of *Tursiops *individuals were examined in this study, further studies including more samples of the two *Tursiops *species should be performed in the future to confirm this.

Introgression could be the third explanation for the anomalous PCR amplification pattern. Numerous cases of dolphin hybridization both in captivity and in the wild [91-94] have been reported. It is reasonable that insertion might have occurred only in the genome of *Tursiops*, however introgression between *Tursiops *and *Stenella *may have taken place at some time, which may explain the unexpected polymorphism of Turt164 between them (Figure 6B).

## Conclusions

A series of additional SINEs were identified to support the monophyly of the order Cetacea as well as Odontoceti, Delphinoidea, and Delphinidae. Especially, several novel SINEs were found to differentiate Delphinidae with other two Delphinoidea families (i.e. Monodontidae and Phocoenidae), whereas the sister group relationship of Monodontidae and Phocoenidae with exclusion of Delphinidae was revealed by the SINE-flanking sequences. Furthermore, members of classical river dolphins and the genera *Tursiops *and *Stenella *were found to be paraphyletic. Estimates of divergence times based on the flanking regions of 12 retroposed elements using a relaxed-clock Bayesian approach furthered our understanding of the rapid radiation events in cetacean evolution. Interestingly, potential ancestral SINE polymorphisms and incomplete lineage sorting in Delphinidae were detected. Although a few loci are potentially anomalous, this study still demonstrated that SINE-based approach is a powerful tool in phylogenetic studies. Identifying additional novel SINE elements that resolve the relationships in the superfamily Delphinoidea and family Delphinidae will be important steps forward in completely resolving the cetacean phylogenetic relationships in the future.

## Methods

### DNA samples and location

Fifteen cetacean species (13 odontocetes and 2 mysticetes, Table 2.) were examined in this study, using hippopotamus as an outgroup. Because all the muscle tissues used in our study were collected from the incidentally killed or stranded dead individuals, no ethical approval is necessary in such cases. All tissue samples were subsequently frozen at -20°C. The voucher specimens were preserved at Nanjing Normal University. Total genomic DNA from muscle tissues was extracted with a standard phenol/chloroform procedure followed by ethanol precipitation [95]. For blood, we used the DNAeasy Blood Extraction Kit (Qiagen) in a separate laboratory facility.

**Table 2:** Samples used in this study.

Order	Suborder	Superfamily	Family	Scientific name	Common name	sampling location
**Cetacea**	**Odontoceti**	**Delphinoidea**	**Delphinidae**	*Tursiops aduncus*	Indo-Pacific	Dongshan, Fujian
					bottlenose dolphin	Province, China
				*Tursiops truncatus *	Common bottlenose	Polar and Oceanic
					dolphin	Park, Shandong
						Province, China
				*Delphinus capensis *	Long-beaked	Leqing, Zhejiang
					common dolphin	Province, China
				*Stenella coeruleoalba *	Striped dolphin	Dongshan, Fujian
						Province, China
				*Stenella attenuata *	Pantropical spotted	Dongshan, Fujian
					dolphin	Province, China
				*Sousa chinensis*	Indo-Pacific	Xiamen, Fujian
					humpbacked dolphin	Province, China
				*Grampus griseus *	Risso's dolphin	Dongshan, Fujian
						Province, China
			**Monodontidae**	*Delphinapterus leucas*	Beluga, white whale	Polar and Oceanic
						Park, Shandong
						Province, China
			**Phocoenidae**	*Nephocaena phocaenoides*	Finless porpoise	Nanjing, Jiangsu
						Province, China
		**Lipotidea**	**Lipotidae**	*Lipotes vexillifer *	Yangtze river	Jiangyin, Jiangsu
					dolphin	Province, China
		**Platanistoidea**	**Platanistidae**	*Platanista gangetica *	South Asian river	
					dolphin	
		**Ziphioidea**	**Ziphiidae**	*Mesoplodon ginkgodens*	Ginkgo-toothed beaked whale	Lvsi, Jiangsu
						Province, China
		**Physeteroidea**	**Kogiidae**	*Kogia sima*	Dwarf sperm whale	Xiamen, Fujian
						Province, China
	**Mysticeti**		**Balaenopteridae**	*Balaenoptera acutorostrata*	Common minke whale	Zhoushan, Zhejiang
						Province, China
				*Balaenoptera omurai*	Omura's whale	Weizhou Iland, Bei hai,
						Guangxi Province, China
**Artiodactyla**			**Hippopotamidae**	*Hippopotamus amphibius*	Hippopotamus	Shanghai zoo, Shanghai
						Province, China

### Strategies to identify novel SINE elements

Three different procedures were applied to isolate and characterize novel phylogenetically informative SINEs from cetaceans.

### Strategy 1

Considering that typical SINEs are often present in numbers that exceed 10^4 ^copies per genome, a sufficient amount of SINE sequences can usually be gained with 60 kbp genomic sequence data. In order to identify novel SINEs in the Indo-Pacific bottlenose dolphin, we used the strategy suggested by Okada et al. [96]. Genomic libraries were constructed for *T. aduncus *(Indo-Pacific bottlenose dolphin). Genomic DNA was first digested by *Hin*dIII, and then DNA fragments with the size of 1.5-2.5 kb were cut out of the gel and purified using QIAquick Gel Extraction Kit (QIAGEN). The purified DNA fragments were ligated into the plasmid vector pUC118 *Hin*dIII/*BAP *(TaKaRa) at 16°C overnight. Aliquots of the ligation reactions were transformed into *Escherichia coli *Top10 competent cells and plated for blue/white selection on media containing X-gal and IPTG. White clones were chosen, isolated, purified, and the inserts were then sequenced and analyzed employing an ABI PRISM 310 Automated Genetic Analyzer (Applied Biosystems, Foster City, CA) with universal (forward and reverse) M13 primers under the instruction of the BigDye Terminator Cycle Sequencing Ready Reaction Kit (Applied Biosystems). 62 kb of genomic sequence data of the Indo-Pacific bottlenose dolphin were randomly sequenced. To find SINEs among these sequences, we aligned these sequences using CLUSTAL X [97] and performed a RepeatMasker search using the RepeatMasker software (Smit & Green, Repeat Masker at http://ftp.genome.Washington.edu/RM/RepeatMasker.html). As most SINEs are derived from tRNA genes, we also performed a local Blast search against all published tRNA-genes. Using this procedure, we discovered 12 copies of tRNA-derived SINEs.

### Strategy 2

In order to further identify novel SINEs in the genome of cetaceans, we used the strategy suggested by Chen and Yang [98]. The genomic libraries were constructed for long-beaked common dolphin, striped dolphin, Indo-Pacific humpbacked dolphin, Risso's dolphin and finless porpoise. About three thousands colonies were screened for each species. Clones identified by nonradioactive southern blotting based on digoxigenin-labeling system were sequenced. With this strategy, 25 informative SINEs that inserted into unique genomic loci during evolution were isolated and characterized.

### Strategy 3

To extract potential novel SINEs from GenBank entries, we downloaded sequence data of about 1.8 million bases for the common bottlenose dolphin from the National Institutes of Health Intramural Sequencing Center at http://asia.ensembl.org/Tursiops_truncatus/Info/Index. To identify SINEs from these sequences, we developed a computer-based search profile in the C programming language that extracts sequences of 100 to 500 nt flanked by 8-nt to 25-nt perfect repeats. About 501 corresponding sequences could be extracted from the common bottlenose dolphin sequences. We subsequently used the local version of RepeatMasker (Smit & Green, Repeat Masker at http://ftp.genome.Washington.edu/RM/RepeatMasker.html) containing a specific library comprising all CHR-1 and CHR-2 subfamily consensus sequences to scan for novel SINEs. We also performed a local Blast search against all published SINEs isolated from the cetacean genomes. In the end, we found 182 novel copies of tRNA-derived SINE element flanked with perfect direct repeats (DRs).

### PCR amplification

To examine the presence or absence of a SINE unit at orthologous in various species, we designed and synthesized a pair of primers that flanked the unit based on the novel SINE loci (Additional file 5). PCR was performed with these primer sets for each SINE locus using cetacean and hippopotamus DNAs as templates. All amplification reactions were conducted on a BioRAD PTC-200 using 2×EasyTaq PCR SuperMix (TransGen Biotech) under the profile: 30 cycles at 93°C for 5 min, 93°C for 1 min, 53°C-59°C for 1 min, and 72°C for 1 min, followed by a 10-min extension at 72°C. The PCR products were electrophoresed in a 1.5% agarose gel and visualized under UV irradiation. Longer products indicated the presence of the SINE, whereas shorter products indicated the absence of the SINE. To confirm the presence or absence of a SINE at the loci, PCR products were sequenced employing an ABI PRISM 310 or 3700 system with bi-directional primers.

### Sequence alignment and phylogenetic analyses

All amplified sequences were analyzed and compared with the GenBank-NCBI database using the BLAST network service (http://www.ncbi.nlm.nih.gov/BLAST/). Multiple sequence alignments were performed by using CLUSTAL X [97] and manually adjusted in GeneDoc. For phylogenetic analysis, the SINE insertion data were compiled into the data matrix, in which SINE absence was coded as 0, and SINE presence was coded as 1 (see Additional file 2). In case where a PCR band was invisible or PCR was not performed, the character state was coded as missing (denoted with '?'). The resultant data matrix were applied to PAUP* (ver. 4. 0b10; [99]) for reconstruction of a strict consensus parsimony tree. The analysis was carried out under ''IRREV.UP'' option, regarding '0' as the ancestral state. Newly obtained sequences data have been deposited in GenBank database (accession numbers JN120481-JN120757).

**Figure 7:**
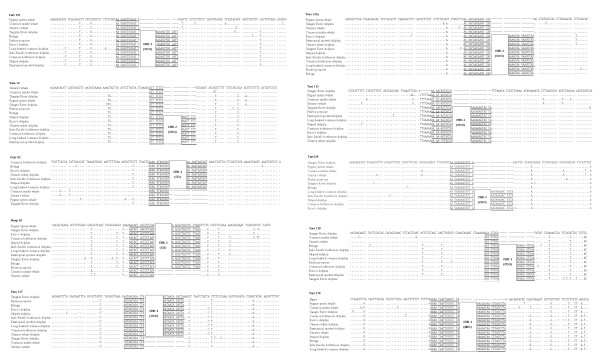
**Concatenations of parts of sequences of the 10 representative SINE loci**. The name of the SINE family as well as its subfamily is indicated in a bold box (CD, Cetacean deletions; CDO, Cetacean deletion Odontoceti; MDI, Middle deletion type I) [16]. The dots indicate nucleotides identical to the consensus sequence at the top. Putative flanking direct repeats are underlined.

In addition, for phylogenetic reconstructions using the flanking regions of 12 retroposed elements, the retroposed elements were entirely removed from the concatenation to make subsequent phylogenetic inferences fully independent of the retroposed insertions, excluding ambiguously aligned sites and highly gapped regions (Figure 7). Bayesian phylogenetic analyses of the concatenated SINE flanking sequence data set (3771 nucleotides in total for each species) were implemented using MrBayes 3.1.2 [100]. Two concurrent runs of one cold and 3 heated Metropolis-coupled Markov chains Monte Carlo (MCMCMC) were launched from random starting points. For DNA sequence alignments, Modeltest 3.7 [101] was employed to choose optimal models for the partition according to the AIC [102]. The 4 MCMCMC were simultaneously run for 20, 000, 000 generations using the program default parameters and trees were sampled every 1000 generations, and the stationarity of the likelihood scores of sampled trees was checked in Tracer 1.4 [103]. Bayesian posterior probabilities (PP) were obtained from the 50% majority-rule consensus of the post burn-in trees sampled at stationarity after removing the first 10% of trees as the "burn-in" stage.

### Molecular divergence estimates

Although SINE insertions allow one to construct tree topologies, they cannot be used for reliable calculation of relative branch lengths without the potential to model amplification rates of SINE markers over time. SINE-flanking sequences, however, may potentially be used for dating historical retropositional events that diagnose common ancestry, because of the probable neutral nature of evolution in nonfunctional regions of the genome [104]. Here, estimation of divergence times was conducted using the flanking regions of 12 retroposed elements with uncorrelated lognormal model, as implemented in BEAST v 1.6 [105]. Age estimates were obtained using the lognormal distribution, with the following fossils as calibration age constraints. The age of the Cetacea-Hippopotamidae split was calibrated using the Ypresian (Eocene: 55.8-48.6 Ma) fossil *Pakicetus *[24,106] with standard deviation (SD) = 1.2. Crown Cetacea was calibrated based on the earliest record of mysticete from the Eocene/Oligocene boundary [79] (33.5-40 Ma, 1.138 SD). The age of the basal of the crown Odontoceti was calibrated using the oldest physeterid: the late Oligocene *Ferecetotherium *[107] (23.7-30 Ma, 1.119 SD). And the age of Phocenidae-Monodontidae split was established based on the oldest Phocoenid, *Salumiphocaena stocktoni *[1982] (10-11.2 Ma, 1.138 SD). The BEAST analysis was executed for 20, 000, 000 generations with a random starting tree, birth-death default priors sampled every 1000 generations. Results were examined using Tracer 1.4 [103] to evaluate stationarity, and the first 10% of trees were discarded as burn-in.

## Authors' contributions

GY and ZC designed the study. ZC carried out the experiments, performed the data analyses and prepared the draft of the manuscript. SX helped to perform the analyses and improve the manuscript. KZ helped to improve the manuscript. GY helped to perform the data analyses and improve the manuscript. All authors read and approved the final manuscript.

## Supplementary Material

Additional file 1 **Electrophoretic gel patterns of PCR products for the SINE loci analyzed in this study**. Bands indicating the presence of the SINE are shown by black arrowheads, whereas gray arrowheads show those that indicate SINE absence. Loci are assigned alphabetically from A to J according to the clade on the phylogenetic tree shown in Figure 4. The species are numbered as follows: 1, Striped dolphin; 2, Risso's dolphin; 3, Indo-Pacific bottlenose dolphin; 4, Common bottlenose dolphin; 5, Long-beaked common dolphin; 6, Chinese white dolphin; 7, Pantropical spotted dolphin; 8, Beluga; 9, Finless porpoise; 10, Yangtze River dolphin; 11, Ginkgo-toothed beaked whale; 12, Ganges River dolphin; 13, Pygmy sperm whale; 14, Omura's whale; 15, Common minke whale; 16, hippopotamus. Click here for file

Additional file 2 **Data matrix showing the character states for the loci isolated in the present study**. 0 = absence, 1 = presence,? = missing. The descriptions of each locus and taxa analyzed in this study are shown in the boxes. Click here for file

Additional file 3 **Alignments of sequences for loci Stec35 (A) and the two different SINE insertions (B)**. Dots indicate nucleotides identical to the consensus sequence at the top. The name of the SINE family as well as the two different SINEs are indicated in a bold box. The line above the sequences represents the tRNA-related region of the SINE. Box A and Box B promoters for RNA Polymerase III are boxed and highlighted. Putative flanking direct repeats are underlined. Click here for file

Additional file 4 **Alignments of sequences for loci Turt164 (A) (including Band A and Band B) and the four SINE insertions amplified in the four species in this study (B)**. Dots indicate nucleotides identical to the consensus sequence at the top. The name of the SINE family as well as its subfamily is indicated in a bold box. The line above the sequences represents the tRNA-related region of the SINE. Box A and Box B promoters for RNA Polymerase III are boxed and highlighted. Putative flanking direct repeats are underlined. Click here for file

Additional file 5 **Primers used in this study**. Click here for file
