# Supplementary figures and images for: Whale phylogeny and rapid radiation events revealed using novel retroposed elements and their flanking sequences
Source: BMC Evol Biol. 2011 Oct 27;11:314. doi: 10.1186/1471-2148-11-314 (PMC3219603; doi:10.1186/1471-2148-11-314)

## Slide 1
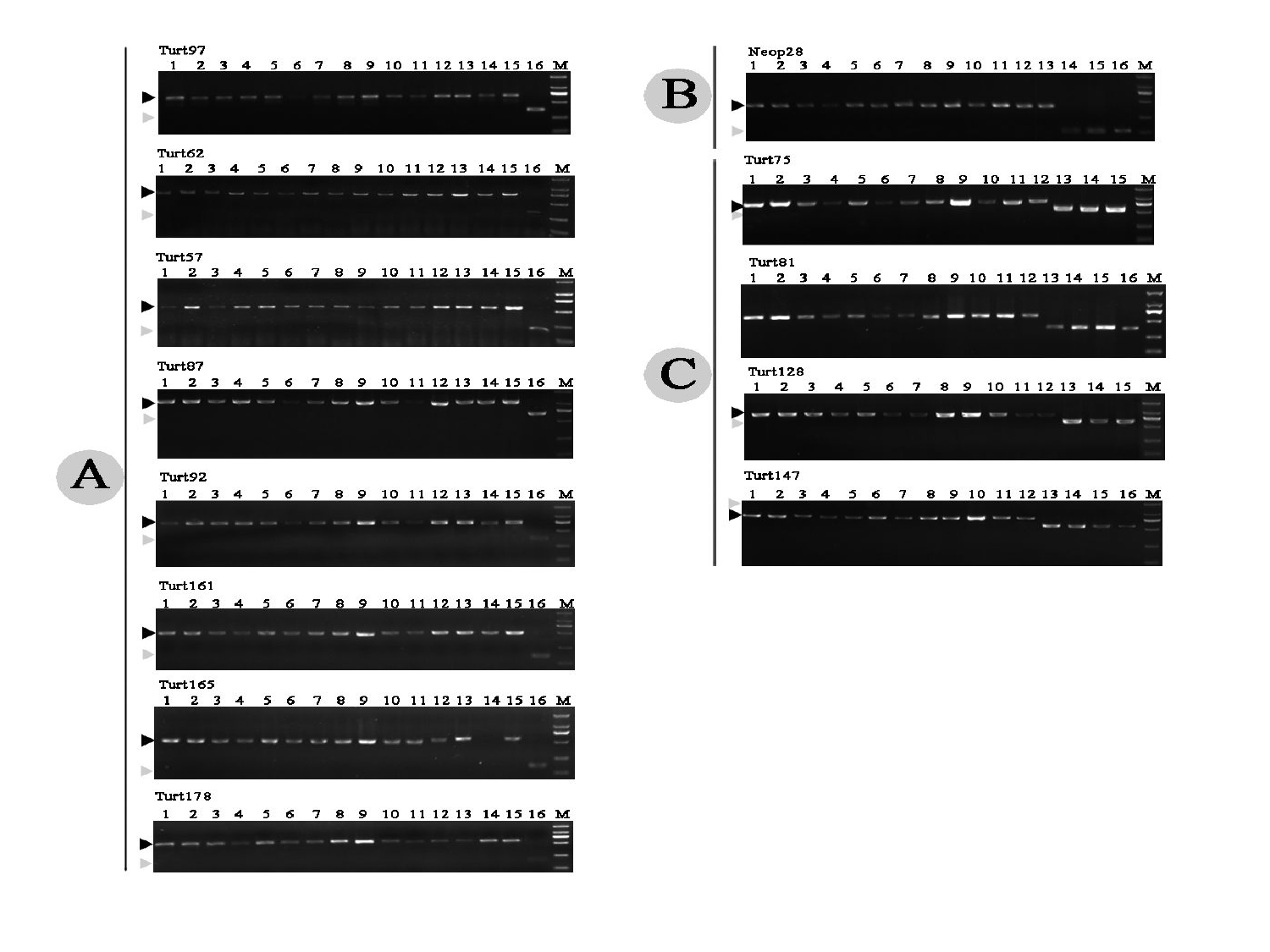

#

## Slide 2
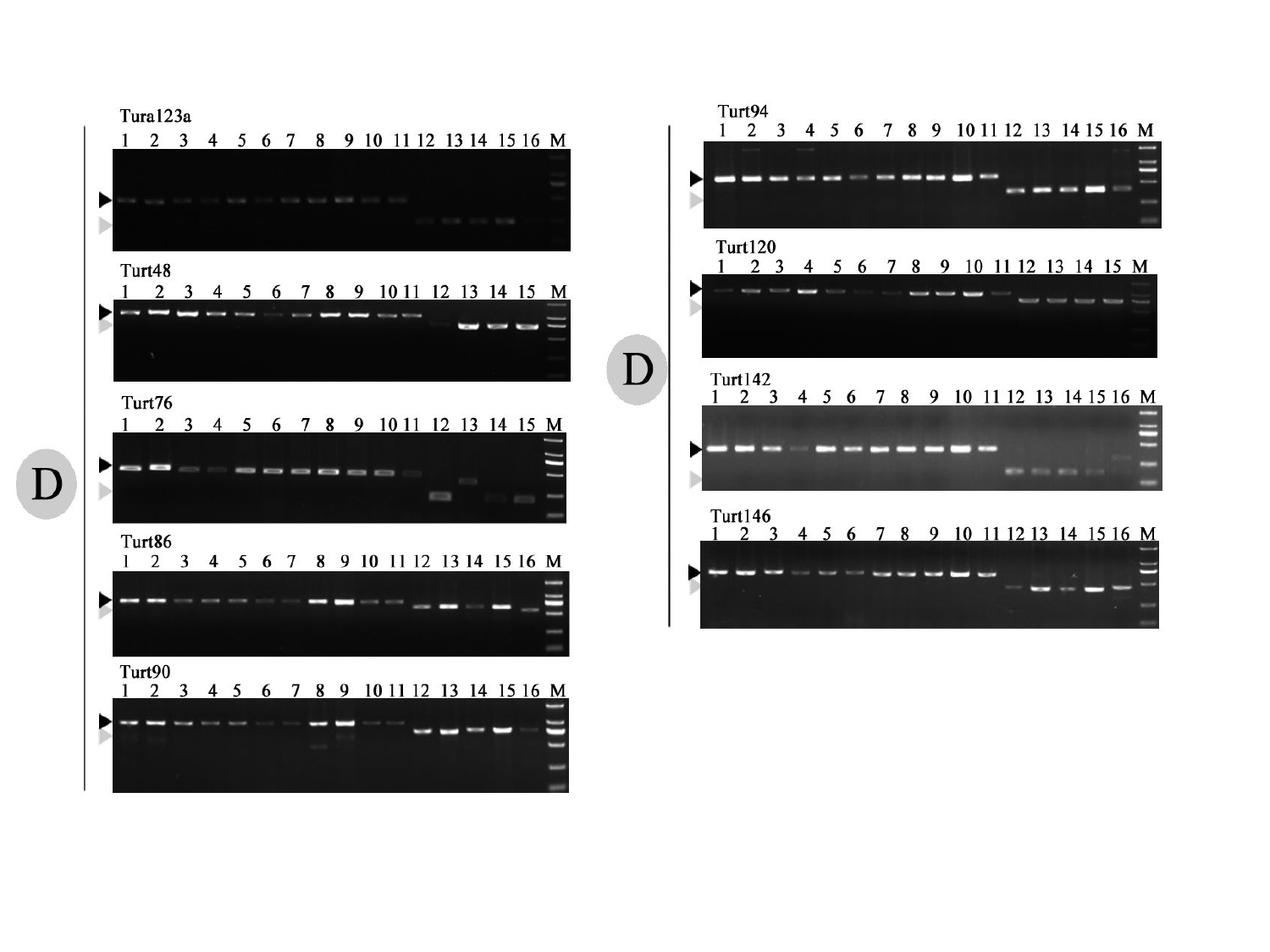

#

## Slide 3
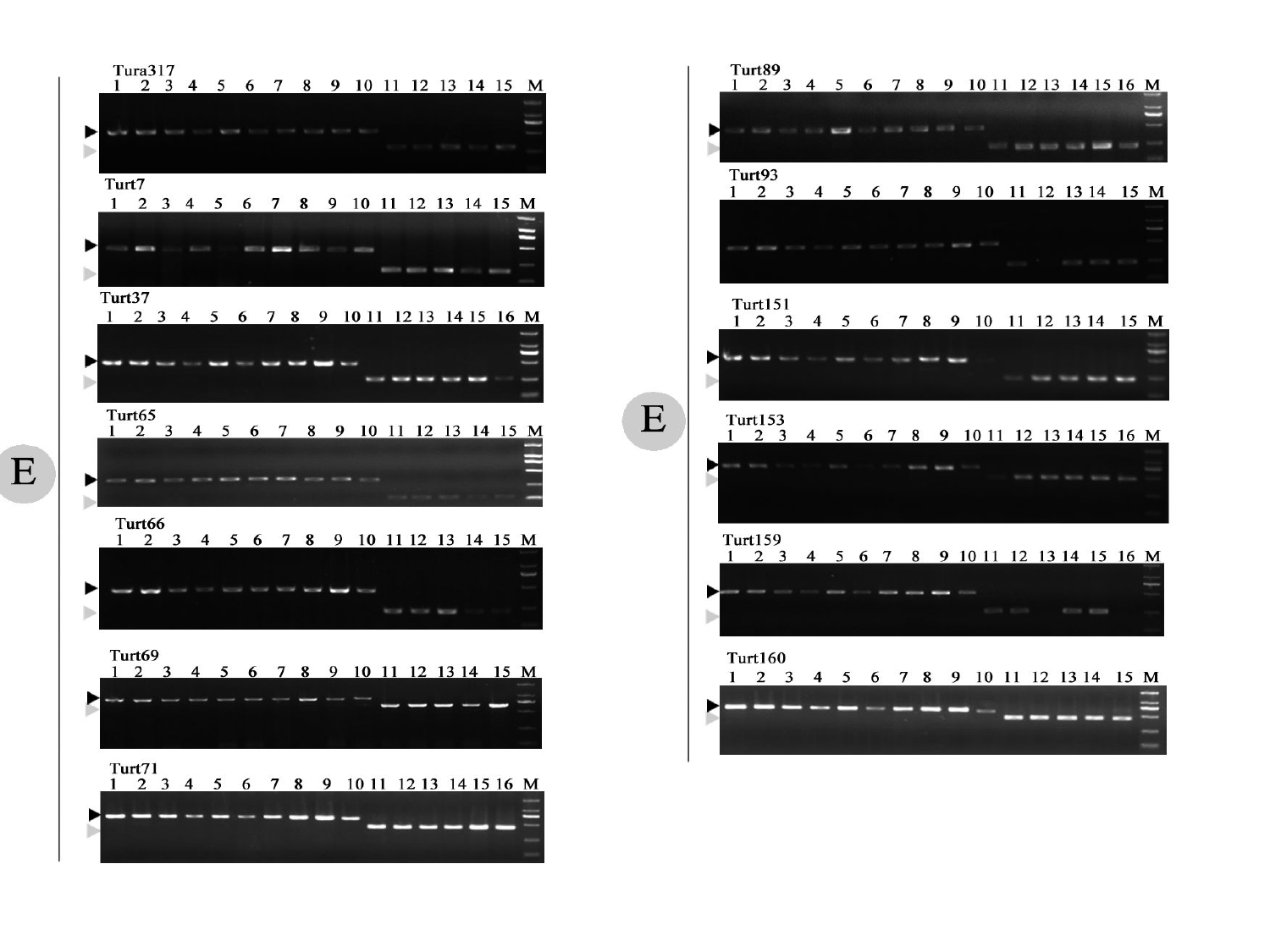

#

## Slide 4
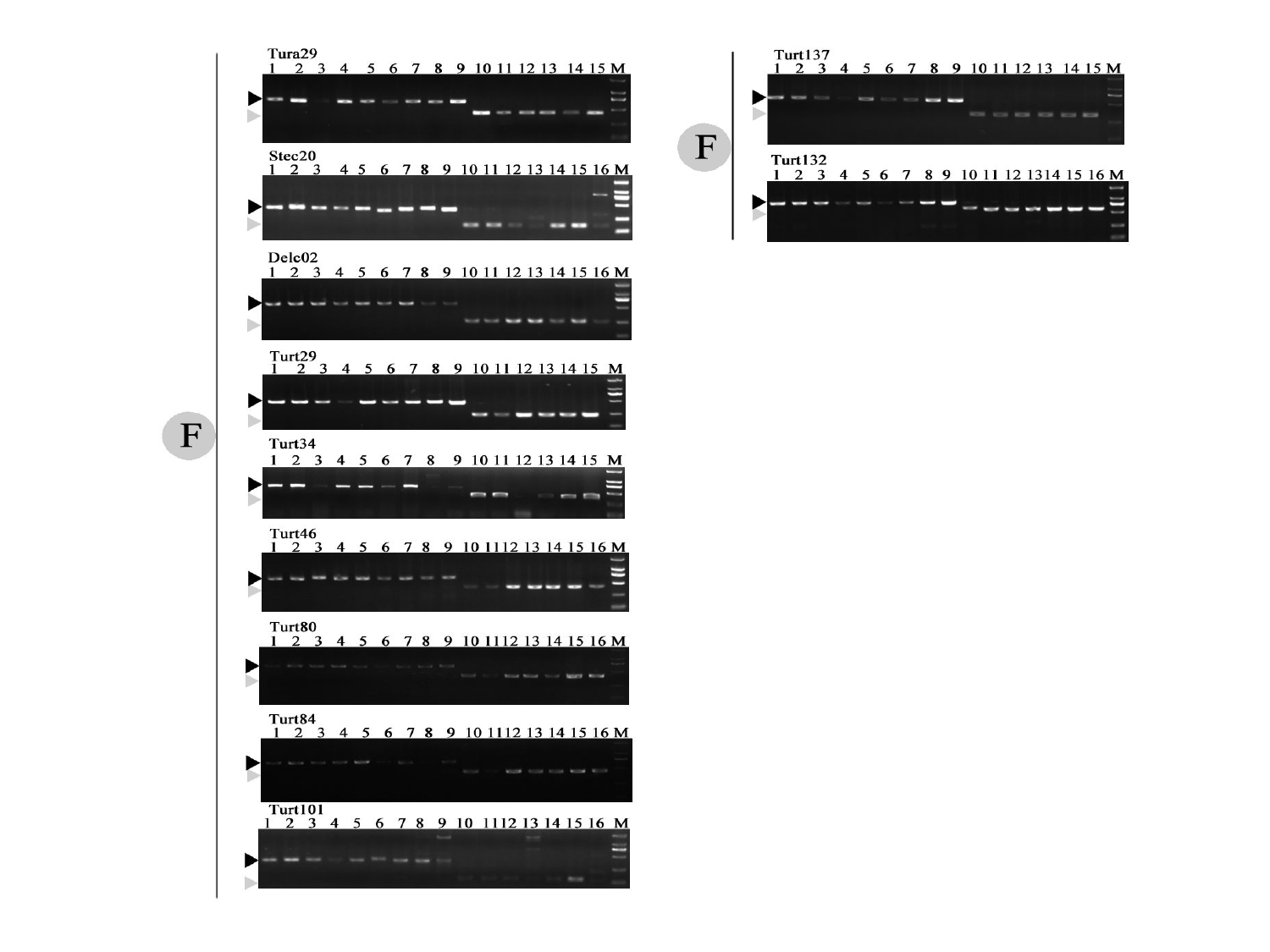

#

## Slide 5
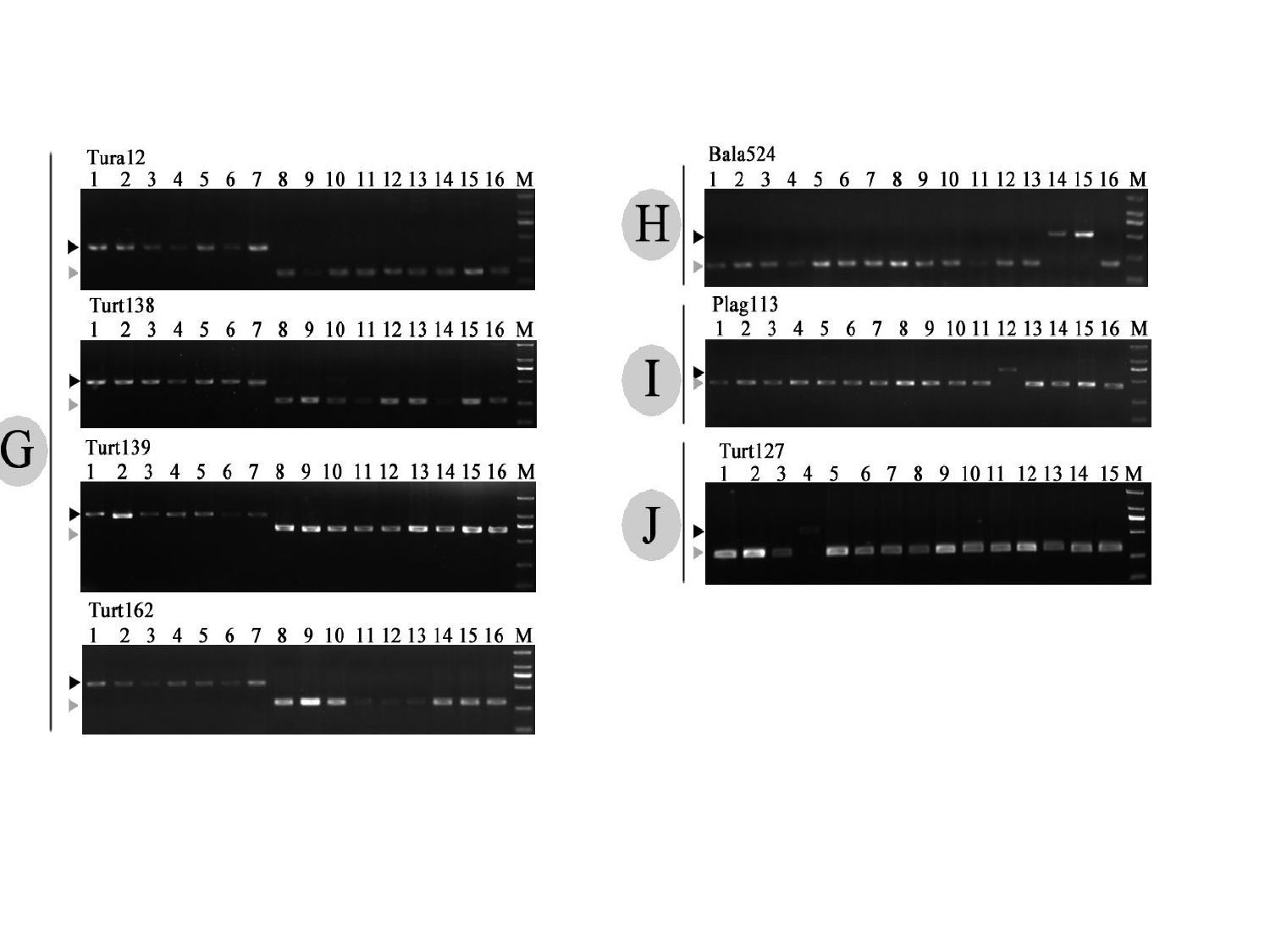

#

Supplement: Additional file 1 — Electrophoretic gel patterns of PCR products for the SINE loci analyzed in this study. Bands indicating the presence of the SINE are shown by black arrowheads, whereas gray arrowheads show those that indicate SINE absence. Loci are assigned alphabetically from A to J according to the clade on the phylogenetic tree shown in Figure 4. The species are numbered as follows: 1, Striped dolphin; 2, Risso's dolphin; 3, Indo-Pacific bottlenose dolphin; 4, Common bottlenose dolphin; 5, Long-beaked common dolphin; 6, Chinese white dolphin; 7, Pantropical spotted dolphin; 8, Beluga; 9, Finless porpoise; 10, Yangtze River dolphin; 11, Ginkgo-toothed beaked whale; 12, Ganges River dolphin; 13, Pygmy sperm whale; 14, Omura's whale; 15, Common minke whale; 16, hippopotamus. [file 1471-2148-11-314-S1.PPT]

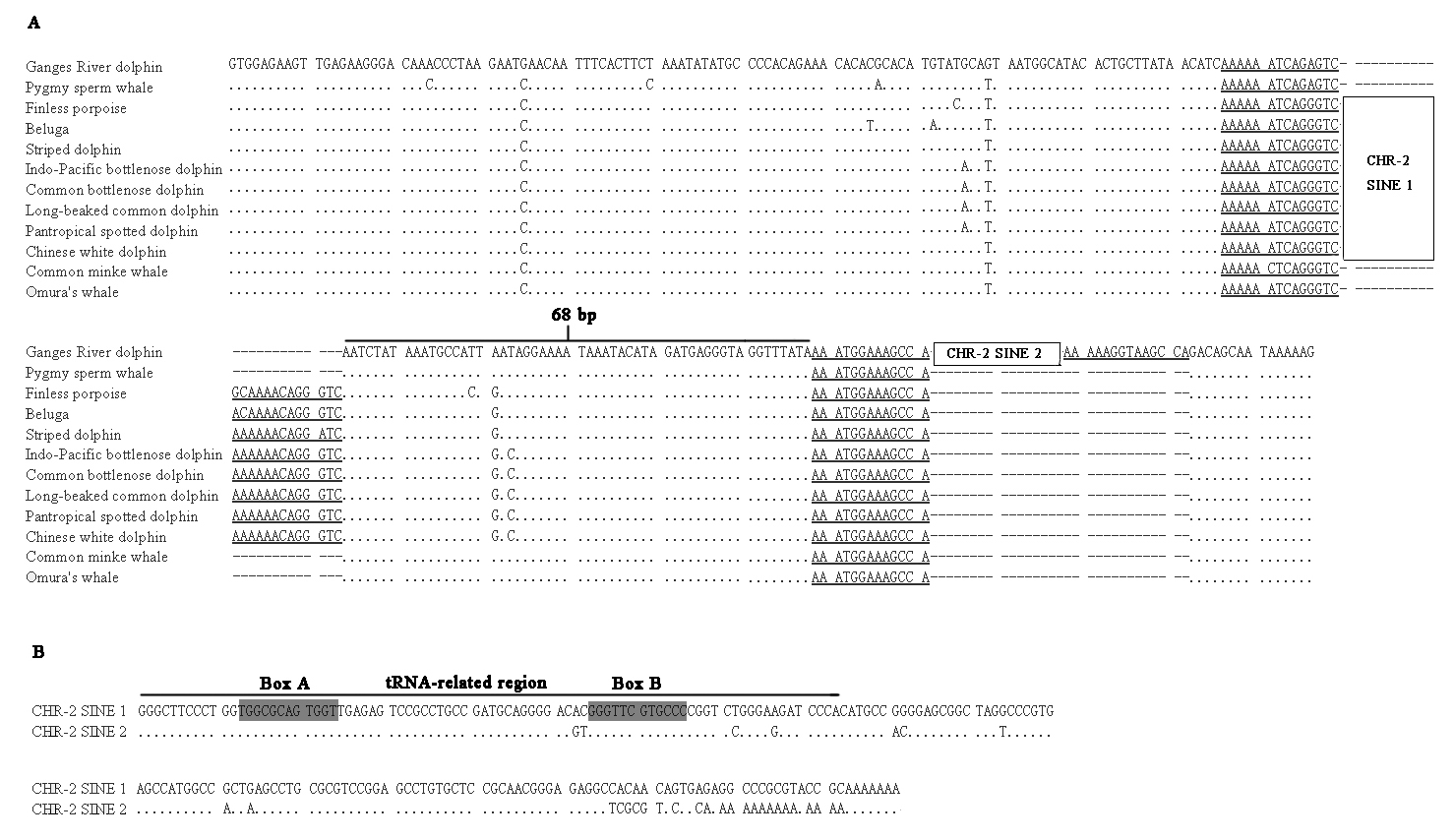

Supplement: Additional file 3 — Alignments of sequences for loci Stec35 (A) and the two different SINE insertions (B). Dots indicate nucleotides identical to the consensus sequence at the top. The name of the SINE family as well as the two different SINEs are indicated in a bold box. The line above the sequences represents the tRNA-related region of the SINE. Box A and Box B promoters for RNA Polymerase III are boxed and highlighted. Putative flanking direct repeats are underlined. [file 1471-2148-11-314-S3.JPEG]

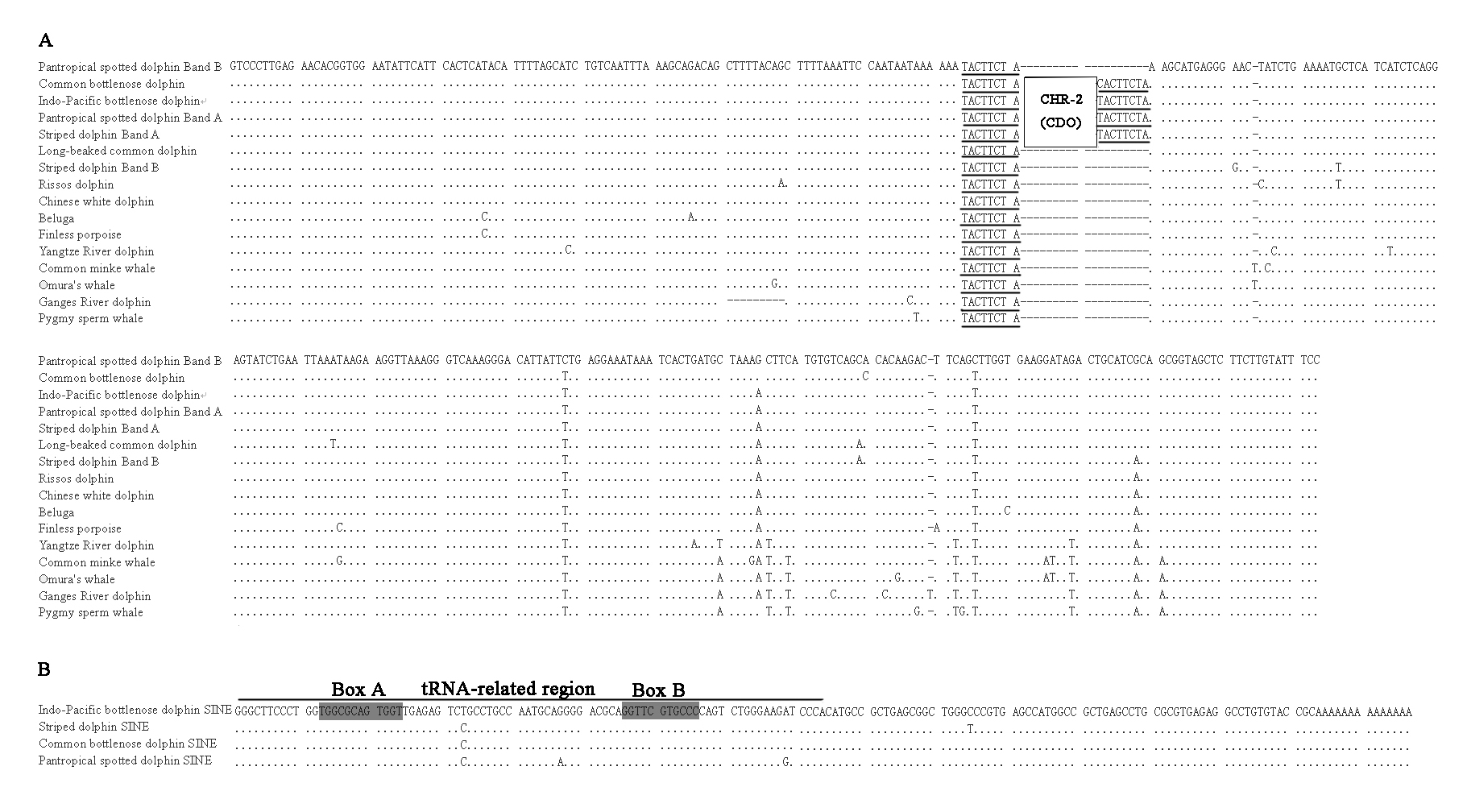

Supplement: Additional file 4 — Alignments of sequences for loci Turt164 (A) (including Band A and Band B) and the four SINE insertions amplified in the four species in this study (B). Dots indicate nucleotides identical to the consensus sequence at the top. The name of the SINE family as well as its subfamily is indicated in a bold box. The line above the sequences represents the tRNA-related region of the SINE. Box A and Box B promoters for RNA Polymerase III are boxed and highlighted. Putative flanking direct repeats are underlined. [file 1471-2148-11-314-S4.JPEG]
