# Supplementary material for: Whale phylogeny and rapid radiation events revealed using novel retroposed elements and their flanking sequences
Source: BMC Evol Biol. 2011 Oct 27;11:314. doi: 10.1186/1471-2148-11-314 (PMC3219603; doi:10.1186/1471-2148-11-314)
Supplement: Additional file 2 — Data matrix showing the character states for the loci isolated in the present study. 0 = absence, 1 = presence,? = missing. The descriptions of each locus and taxa analyzed in this study are shown in the boxes. [file 1471-2148-11-314-S2.DOC]

Locus

Species 1 2 3 4 5 6 7 8 9 10 11 12 13 14 15 16 17 18 19 20 21 22 23 24 25 26 27 28 29 30 31 32 33 34 35 36 37 38 39 40 41 42 43 44 45 46 47 48 49 50 51 52 53 54 55 56 57 58 59 60 61 62 63 64

a 1 1 1 1 1 1 1 1 1 1 0 1 1 1 1 1 1 1 1 1 1 1 1 1 1 1 1 1 1 1 1 1 1 1 1 1 1 1 1 1 1 0 1 0 1 1 1 1 1 1 1 1 1 1 1 1 1 1 1 1 1 1 1 0

b 1 1 1 1 1 1 1 1 1 1 0 1 1 1 1 1 1 1 1 1 1 1 1 1 1 1 1 1 1 1 1 1 1 1 1 1 1 1 1 1 1 0 1 0 1 1 1 1 1 1 1 1 1 1 1 1 1 1 1 1 1 1 1 0

c 1 1 1 1 1 1 1 1 1 1 0 1 1 1 1 1 1 1 1 1 1 1 1 1 1 1 1 1 1 1 1 1 1 1 1 1 1 1 1 1 1 0 1 0 1 1 1 1 1 1 1 1 1 1 1 1 1 1 1 1 1 1 1 0

d 1 1 1 1 1 1 1 1 1 1 0 1 1 1 1 1 1 1 1 1 1 1 1 1 1 1 1 1 1 1 1 1 1 1 1 1 1 1 1 1 1 0 1 1 1 1 1 1 1 1 1 1 1 1 1 1 1 1 1 1 1 1 1 0

e 1 1 1 1 1 1 1 1 1 1 0 1 1 1 1 1 1 1 1 1 1 1 1 1 1 1 1 1 1 1 1 1 1 1 1 1 1 1 1 1 1 0 1 0 1 1 1 1 1 1 1 1 1 1 1 1 1 1 1 1 1 1 1 0

f 1 1 1 1 1 1 1 1 1 1 0 1 1 1 1 1 1 1 1 1 1 1 1 1 1 1 1 1 1 1 1 1 1 1 1 1 1 1 1 1 1 0 1 0 1 1 1 1 1 1 1 1 1 1 1 1 1 1 1 1 1 1 1 0

g 1 1 1 1 1 1 1 1 1 1 0 1 1 1 1 1 1 1 1 1 1 1 1 1 1 1 1 1 1 1 1 1 1 1 1 1 1 1 1 1 1 0 1 0 1 1 1 1 1 1 1 1 1 1 1 1 1 1 1 1 1 1 1 0

h 0 1 1 1 1 1 1 1 1 1 0 1 1 1 ? 1 1 1 1 1 1 1 1 1 1 1 1 1 1 1 1 1 1 1 1 1 1 1 1 1 1 0 1 0 1 0 1 1 1 1 1 0 1 1 1 1 1 1 1 1 1 0 1 0

i 0 1 1 1 1 1 1 1 1 1 0 1 1 1 1 1 1 1 1 1 1 1 1 1 1 1 1 1 1 1 1 1 1 1 1 1 1 1 1 1 1 0 1 0 1 0 1 1 1 1 1 0 1 1 1 1 1 1 1 1 1 0 1 0

j 0 0 1 1 1 0 1 0 1 1 0 0 1 1 0 0 1 ? 1 1 1 1 1 1 1 1 1 1 1 1 0 0 1 1 ? 1 1 1 0 1 1 0 1 0 1 0 1 0 0 1 1 0 1 1 1 1 1 1 1 1 1 0 ? 0

k 0 0 1 0 1 0 1 0 0 1 0 0 ? 0 0 0 1 ? 1 0 1 1 0 0 0 1 1 0 1 1 0 0 1 1 ? 1 0 1 0 ? 1 0 1 0 ? 0 1 0 0 1 1 0 1 1 0 0 0 0 1 1 ? 0 1 0

l 0 0 0 0 1 0 0 0 0 1 0 0 0 0 ? 0 0 ? 1 0 1 1 0 0 0 1 0 0 0 1 0 0 1 0 0 1 ? 0 0 1 1 1 0 0 0 0 1 0 0 1 0 0 0 1 0 0 0 0 1 1 0 0 1 1

m 0 0 0 0 1 0 0 0 0 ? 0 0 0 0 0 0 0 0 1 0 1 1 0 0 0 0 0 0 0 0 0 0 1 0 ? 1 0 0 0 ? 1 0 0 0 0 0 0 0 0 0 0 0 0 0 0 0 0 ? 1 1 0 ? 1 0

n 0 0 0 0 0 0 0 0 0 0 1 0 0 0 0 0 0 0 0 0 1 1 0 0 0 0 0 0 0 0 0 0 1 0 0 1 0 0 0 0 1 0 0 0 0 0 0 0 0 0 0 0 0 0 0 0 0 0 1 1 0 0 1 0

o 0 0 0 0 0 0 0 0 0 0 1 0 0 0 0 0 0 0 0 0 1 1 0 0 0 0 0 0 0 0 0 0 1 0 0 1 0 0 0 0 1 0 0 0 0 0 0 0 0 0 0 0 0 0 0 0 0 0 1 ? 0 0 1 0

p 0 ? 0 ? 0 0 ? 0 ? ? 0 ? 0 0 ? 0 ? ? 0 ? 0 0 ? ? 0 0 ? 0 0 0 0 0 0 0 0 0 ? 0 0 ? 0 0 ? ? 0 0 ? 0 ? ? 0 0 0 0 ? 0 ? ? 0 0 ? 0 0 0

| a: Striped dolphin | i: Finless porpoise |
| --- | --- |
| b: Risso’s dolphin | j: Yangtze River dolphin |
| c: Indo-Pacific bottlenose dolphin | k: Ginkgo-toothed beaked whale |
| d: Common bottlenose dolphin | l: Ganges River dolphin |
| e: Long-beaked common dolphin | m: Pygmy sperm whale |
| f: Chinese white dolphin | n: Omura’s whale |
| g: Pantropical spotted dolphin | o: Common minke whale |
| h: Beluga | p: Hippopotamus |
|  |  |

| 1: Tura12 | 14: Turt37 | 27: Turt76 | 40: Turt96 | 53: Turt142 |
| --- | --- | --- | --- | --- |
| 2: Tura29 | 15: Turt34 | 28: Turt89 | 41: Turt97 | 54: Turt147 |
| 3: Tura123a | 16: Turt46 | 29: Turt86 | 42: Plag113 | 55: Turt151 |
| 4: Tura317 | 17: Turt48 | 30: Turt81 | 43: Turt120 | 56: Turt153 |
| 5: Neop28 | 18: Turt51 | 31: Turt80 | 44: Turt127 | 57: Turt160 |
| 6: Delc02 | 19: Turt61 | 32: Turt84 | 45: Turt125 | 58: Turt159 |
| 7: Stec35 | 20: Turt65 | 33: Turt87 | 46: Turt139 | 59: Turt161 |
| 8: Stec20 | 21: Turt57 | 34: Turt90 | 47: Turt128 | 60: Turt165 |
| 9: Turt7 | 22: Turt62 | 35: Turt91 | 48: Turt132 | 61: Turt169 |
| 10:Turt12 | 23: Turt66 | 36: Turt92 | 49: Turt137 | 62: Turt162 |
| 11:Bala524 | 24: Turt69 | 37: Turt93 | 50: Turt140 | 63: Turt178 |
| 12:Turt29 | 25: Turt71 | 38: Turt94 | 51: Turt146 | 64: Plag35 |
| 13:Turt38 | 26: Turt75 | 39: Turt101 | 52: Turt138 |  |
